# Broken symmetries and the related interface-induced effects at Weyl-system TaAs in proximity of noble metals

**DOI:** 10.1038/s41598-020-71494-w

**Published:** 2020-09-02

**Authors:** Tuhin Kumar Maji, Kumar Vaibhav, Samir Kumar Pal, Debjani Karmakar

**Affiliations:** 1 Department of Chemical Biological and Macromolecular Sciences, S.N. Bose National Centre for Basics Sciences, Salt Lake, Sector 3, Kolkata, 700106 India; 2 Computer Division, Bhabha Atomic Research Centre, Trombay, Mumbai 400085 India; 3 Technical Physics Division, Bhabha Atomic Research Centre, Trombay, Mumbai 400085 India

**Keywords:** Materials science, Physics

## Abstract

Weyl semimetal TaAs, congenially accommodating the massless Weyl fermions, furnishes a platform to observe a spontaneous breaking of either the time-reversal or the inversion symmetry and the concurrent genesis of pairs of Weyl nodes with significant topological durability. Former experimental analysis, which reveals that the near-zero spin-polarization of bulk TaAs, experiences a boost in proximity of point-contacts of non-magnetic metals along with the associated tip-induced superconductivity, provides the impetus to study the large-area stacked interfaces of TaAs with noble metals like Au and Ag. The primary outcomes of the present work can be listed as follows: (1) First-principles calculations on the interfacial systems have manifested an increment of the interface-induced spin-polarization and contact-induced transport spin-polarization of TaAs in proximity of noble metals; (2) In contrast to the single interface, for vertically stacked cases, the broken inversion symmetry of the system introduces a *z*-directional band-dispersion, resulting in an energetically separated series of non-degenerate band crossings. The simultaneous presence of such band-crossings and spin-polarization indicated the coexistence of both broken time reversal and inversion symmetries for metal-semimetal stacked interfaces; (3) quantum transport calculations on different device geometries reveal the importance of contact geometry for spin-transport in TaAs devices. Lateral contacts are found to be more effective in obtaining a uniform spin transport and larger transport spin polarization; (4) the phonon dispersion behaviour of TaAs displays a closure of band-gap with the associated increase of phonon-density of states for the acoustic modes in proximity of lateral contacts of noble metals.

## Introduction

Observation of massless Weyl fermion in topologically non-trivial Weyl semimetal systems like Tantalum and Niobium arsenides and phosphides^1,2^ bridges the long-pending gap between its prediction in high-energy physics and the condensed matter realization as an eminent quasiparticle excitation^3^. Such observation complements the similar discovery of its Dirac^4,5^ or Majorana^6,7^ counterparts in periodic systems.

Crystalline Weyl semimetal (WSM) systems consist of bulk band crossings, named as Weyl nodes, consisting of non-degenerate three-dimensional bands near Fermi-level, in contrast to the two-dimensional band crossings at Dirac points of Graphene^8,9^. Near the nodal point, the low-energy physics is governed by Weyl equations^10^, having solutions as massless Weyl spinors with distinct chirality, as obtained from relativistic field theory^3,11–14^. Realization of Weyl nodes is possible, when the time reversal symmetry (TRS) or inversion symmetry (IS) is broken for a Dirac semi-metal^4,5,15,16^, where a Dirac node can be considered to be composed of two Weyl nodes of opposite chirality^17^. Thus, Weyl nodes in a crystalline system always appear in pairs of opposite chirality at two distinctly separated *k*-points, so that, their annihilation by simple translational symmetry preserving perturbations, are prevented, rendering the topological stability of the system^18^. The hallmark signature of WSM is the presence of Fermi-arcs on the surface, joining two Weyl nodes of opposite chirality^1,2,17,19,20^. The degeneracy associated with Weyl nodes at the 3D Weyl cones in the systems like Cd_3_As_2_^5^, Na_3_Bi^4,21^, Ta and Nb phosphides and arsenides^1,2,17,19,20^ or at the 2D surface states of topological insulators(TI)^6–8^ depends merely on translational symmetry. Even before the discovery of Weyl systems, there was proposal of realization of 3D semimetal phase by alternate vertical stacks of topological insulators and band insulators^22^. For the same stack, Zyunin et al. have explored the outcome of simultaneously broken TRS and IS. While the broken TRS ensures the topological stability of the Weyl nodes by separating them in momentum space but occurring at the same energy, broken IS, without disturbing the topological nature of the state, shifts the Weyl nodes at different energies^23^. Recent discovery of a series of magnetic Weyl semimetals with broken time reversal symmetries widens the horizon of topological semi-metals^8,11,24–27^.

A significant property of WSM is the singularity of Berry curvature (BC) at the Weyl nodes, representing a magnetic monopole in the momentum space with a distinct chirality^28–31^. These nodal points act as a source or a sink of the BC for the positive and negative chirality respectively and thus their pair-occurrence prevents the divergence of BC. Such topological systems are also defined by Chern numbers, representing the integral of BC over any closed 2D manifold at the Fermi-level^5,19^. The Chern number can have a nonzero (zero) value, depending on the inclusion (exclusion) of a Weyl node within the 2D manifold^20^. WSMs are well known to have anomalous nature of their DC transport related to the absence of backscattering and weak antilocalization^19,32,33^, as a consequence of presence of topological surface states having well-defined spin. Another interesting chiral signature of WSM is the chiral anomaly, where, in presence of electric and magnetic fields, the particle number corresponding to a particular valley is not conserved^33^, culminating intervalley pumping of electrons between two nodes of opposite chirality^33–37^.

In addition to the exciting fundamental physics, topological systems like Cd_3_As_2_^38^ and TaAs^39–42^ exhibited mesoscopic superconducting phase and high transport spin polarization in presence of metallic point contacts like Ag. However, regarding the occurrence of superconducting phase in TaAs, debates persist about the nature of the pairing symmetry. Whereas, Wang et al. had proposed unconventional *p*-wave superconductivity^41,43^, Gayen et al. had demonstrated an *s*-wave conventional nature^42^. The unresolved controversies about the fundamental nature of superconductivity and the urge of understanding the interfacial proximity effects have motivated the current investigation of large-area interfaces of Weyl semimetal system TaAs with two well-known noble metals Au and Ag. While proximity effects are well studied for metal–semiconductor junctions^44^, for transfer of topological phase at Graphene/TI(Bi_2_Se_3_) heterostructure^45^ or for conventional insulator/ TI(Bi_2_Se_3_) heterostructure^46^, theoretical study of metal-semimetal proximity effects on the electronic and quantum transport properties are scarce in literature.

In the next section, we brief the computational methodology used for the theoretical investigations. The successive section describes the structures and the underlying symmetry of the bare and stacked interfaces and their respective band structures. The quantum transport properties of the devices with TaAs as channel material and Au/Ag as lateral/vertical contacts are described in the next section. As a next step, phonon dispersions of the lateral interfacial systems are investigated. The last section summarizes the obtained results with a conclusion.

### Computational methodology

The first principles electronic structure of the single and stacked TaAs/Au and TaAs/Ag interfaces are carried out by using the Vienna ab initio simulation package (VASP)^47,48^ with norm-conserving projector augmented wave (PAW) pseudopotentials and generalized gradient approximated (GGA) Perdew-Burke-Ernzerhof (PBE) exchange–correlation functionals^49^ with incorporation of spin–orbit (SO) coupling. For the pseudopotentials, the valence levels for Ta and As consist of 5*p*^6^6*s*^2^5*d*^3^ and 4*s*^2^4*p*^3^ configurations respectively. Van der Waal corrections are incorporated by following the semi-empiricial Grimme DFT-D2 method^50^. The plane-wave cutoff and Monkhorst–Pack *k*-points grid^51^ are set as 500 eV and 7 × 7 × 5 respectively. Ionic relaxations are performed by using conjugate gradient algorithm^52^ with the cutoff for the Hellmann–Feynman force as 0.01 eV/Å.

For the density functional theory (DFT)-coupled quantum transport, we have used the Atomistic Toolkit 15.1 packages^53^, with the GGA-PBE exchange correlation and double-zeta plus polarization (DZP) basis set. For each lateral/vertical interface, ionic optimizations are carried out to relax the interfaces with the real-space energy cutoff as 200 Hartree and the maximum force of 0.01 eV/Å. For quantum transport calculations using the two-probe model, we have used the DFT coupled nonequilibrium Green’s function (NEGF) method. Devices with appropriate channel length of TaAs, nullifying inter-electrode transmission and lateral/vertical contacts of Au/Ag, are constructed after fully relaxing the lateral and vertical interfaces with metals. Two sets of transport calculations are performed after keeping the electron temperatures as 300 K and 5 K. At interfaces of the electrodes and the central region, Dirichlet boundary condition has been employed to ensure the charge neutrality in the source and the drain region. The channel length was optimized to ensure zero contributions from the inter-electrode transmissions. The Monkhorst–Pack *k*-point mesh is sampled with 5 × 5 × 50. The transport properties and the corresponding transmission coefficients are calculated by averaging over a ${k}_{x}\times {k}_{y}$ mesh of 10 $\times$ 10 in a direction perpendicular to the current transmission. Γ-point centred transmission coefficients, perpendicular to the transport axis within the irreducible Brillouin zone (IBZ), are calculated for both the lateral and vertical device geometries.

We have used supercell method to calculate the phonon dispersion and phonon DOS, where the dynamical matrices are calculated over a *q*-point grid 5 × 5 × 5, and the Hamiltonian derivatives are calculated over a *k*-mesh 10 × 10 × 10.

### Structural construction of interfaces

**Figure 1:**
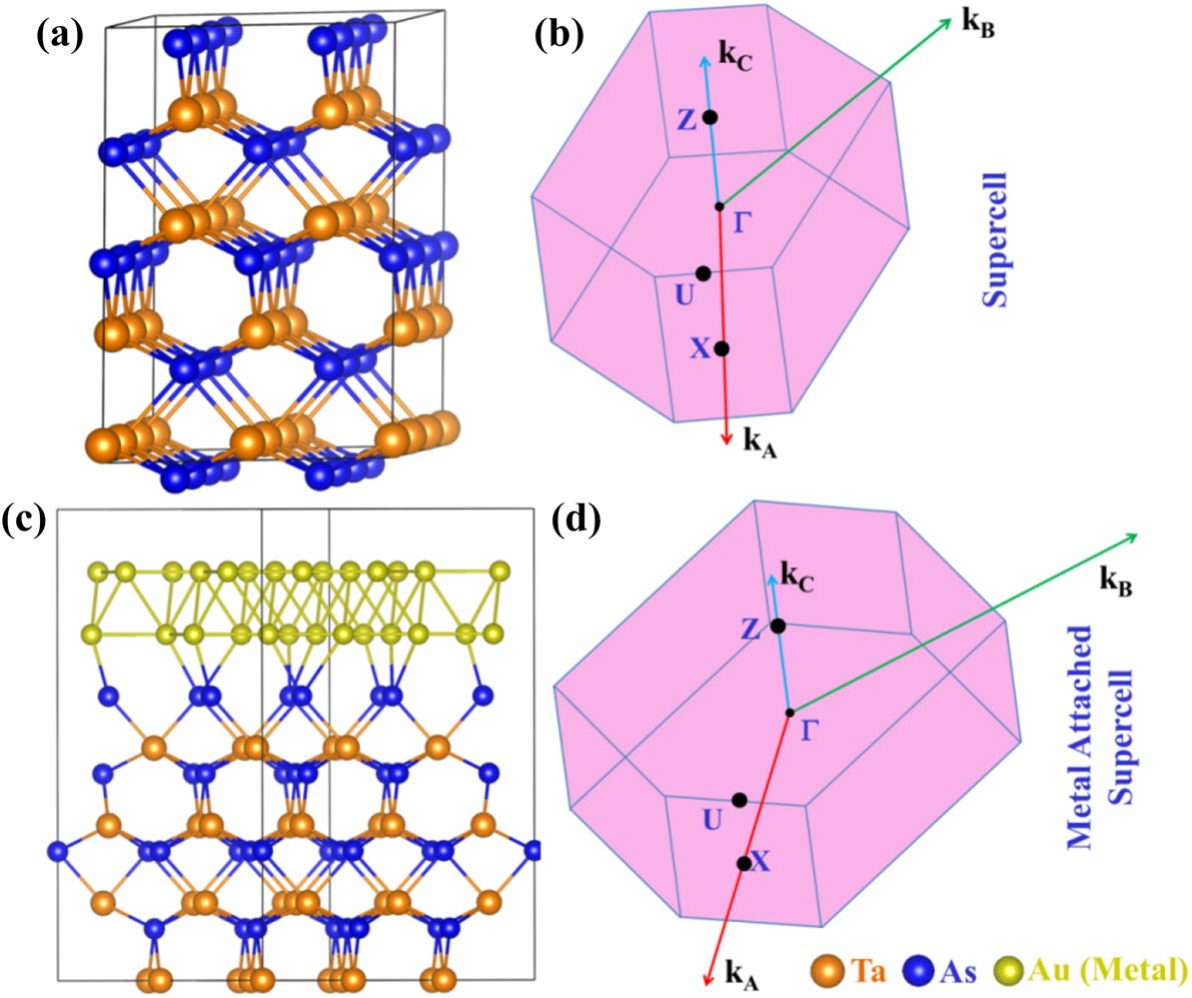
(**a**) Structural image of TaAs supercell in P_1_ symmetry, (**b**) Brillouin Zone and high symmetry points of the supercell, (**c**) structural image of TaAs/Au heterostructure, (**d**) Brillouin Zone and high symmetry points of the heterostructure in P_1_ symmetry. The colour of the corresponding elements are denoted within figure.

Bulk TaAs belongs to a body-centred (BC) tetragonal lattice structure with space group *I4*_*1*_*md* containing two formula units per unit cell. The system lacks an inversion symmetry and for its [001] surface, the *C*_*4*_ rotational symmetry is also broken^1,2,17^. We have constructed the interfaces with the bilayer Au [111] and Ag [111] surfaces with the [3 × 3 × 2] supercell of TaAs. For the vertical interfaces Au–TaAs and Ag–TaAs, the mean interfacial strain is minimized to 1.4% after a mutual rotation between TaAs and metal [111] surface by ~ 29° by following the Co-incident site lattice (CSL) method as implemented in ATK^53,54^. The underlying symmetry of the BC-tetragonal lattice transforms to a triclinic (*P*_1_) one for the interface. Figure 1 depicts the structure of the supercell in *P*_1_ symmetry, the corresponding interfaces and the Brillouin zones with the high-symmetry points for these systems. For TaAs/Au and TaAs/Ag single interface, we have added a vacuum of 15 Å in the *z*-direction to avoid the z-directional periodic replication. For stacked interfaces, the TaAs/metal stack repeats itself along z-direction without any vacuum-induced breaking of translational symmetry.

### Electronic structure of TaAs for triclinic symmetry

**Figure 2:**
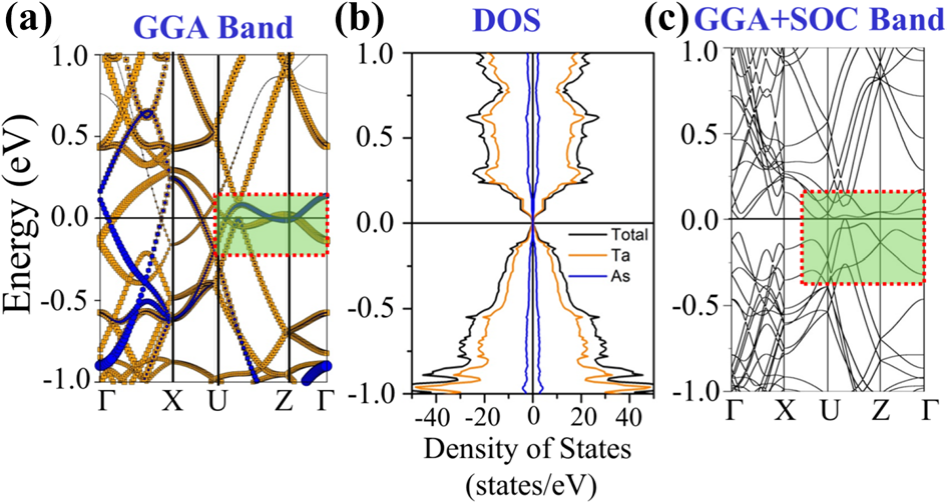
Orbital projected (Ta-d—orange, As-p—blue), (**a**) band-structure and (**b**) spin-up and spin-down density of states of TaAs supercell using GGA, (**c**) GGA + SO gapped band structure of the same supercell.

The orbital projected band structure of TaAs supercell (as depicted in Fig. 1a) along high symmetry directions of the triclinic BZ (Fig. 1b) is depicted in Fig. 2a. There are band-inversions across *E*_F_, with multiple spin-degenerate 3D band-crossings, which along the high-symmetry directions U–Z and Z–Γ, resemble with Weyl-cone features, as highlighted within the red dotted rectangle in Fig. 2a. Ta and As, being in the 3+ and 3− valence states, have one-fifth filled Ta-5*d* (5*d*^2^) and one-third filled As-4*p* (4*p*^2^) valence levels contributing near *E*_F_. Bands near *E*_F_ have highly hybridized Ta-5*d* and As-4*p* character, as can be seen from the Fig. 2a. Figure 2b represents the orbital- projected density of states (DOS). Near *E*_F_, the DOS shows the conventional semi-metal like behavior^55^. Figure 2c shows the expected splitting of Weyl Cones after application of SO-coupling (SOC) producing a fully gapped band structure^17,20^. Near *E*_F_ bands, mostly having Ta-5*d* character, are prone to SOC. With SOC, the spin-degeneracy of the band is lifted, after producing a gapped band-structure except at Kramer’s points^20,56^, representing the non-degenerate band crossings. The band-crossings are now shifted away from high-symmetry line, as highlighted within the red dotted rectangle in Fig. 2c. Therefore, after incorporating SOC, time reversal symmetry remains intact in TaAs. In supplementary materials, we have presented the GGA + U band-structure and DOS after incorporating the Hubbard U and exchange J parameter for the Ta-5*d* orbitals.

**Figure 3:**
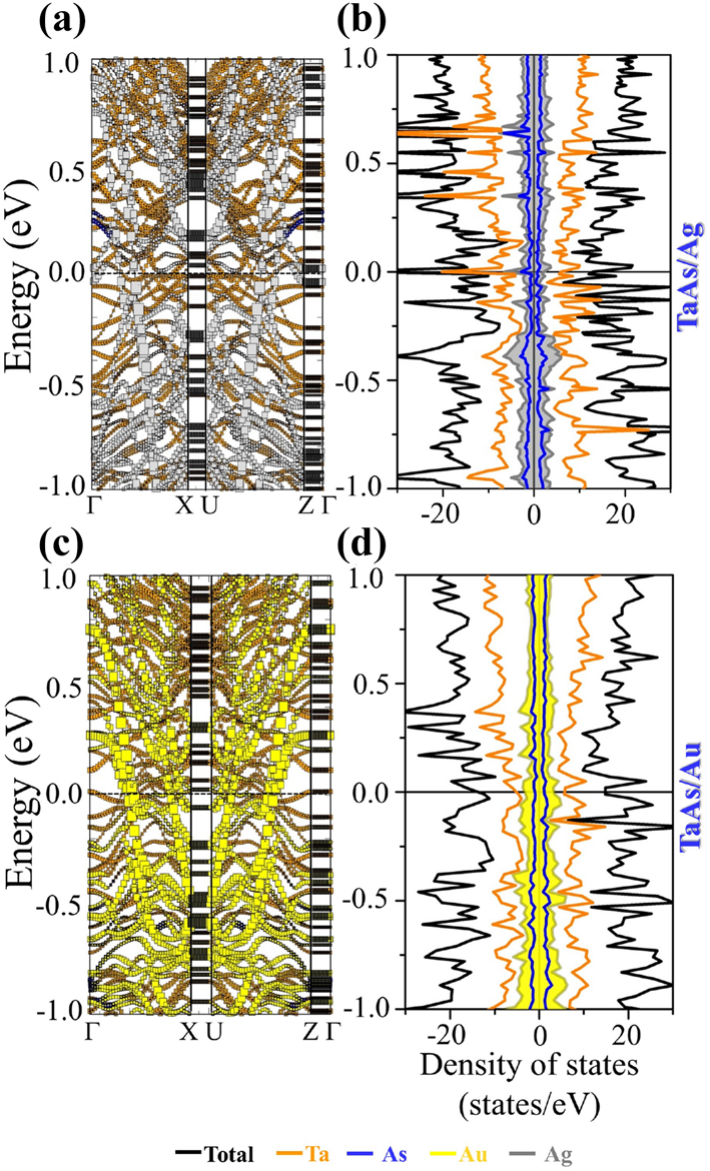
(**a**) Band structure and the corresponding (**b**) orbital Projected spin-up and spin-down DOS of TaAs/Ag single interface with vacuum and (**c**) the band structure and corresponding (**d**) orbital Projected spin-up and spin-down DOS of TaAs/Au single interface with vacuum using GGA. The color coding for orbital projections are: Ta-d—orange, As-p—blue, Ag—grey and Au—yellow.

For TaAs/Ag or TaAs/Au single interfaces, presence of large vacuum destroys the *z*-directional periodicity rendering 2D-like degenerate bands along X–U and Z–Γ, as a result of quantum confinement. The corresponding layer-projected DOS are depicted in Fig. 3b and d respectively. Closer inspection of DOS and band structures indicate the following attributes as a result of the proximity-induced inter-layer charge transfer, viz. (1) vacuum-slab induced quantum confinement, leading to the destruction of the Weyl-cone feature, (2) doping of the TaAs layer underneath metal and (3) spin-polarization of the system as a whole^45,46^. The mutual charge transfer from the delocalized orbitals of Ag/Au (grey/yellow) introduces an *n*-type doping for both the TaAs/Au and TaAs/Ag systems. Pristine TaAs system has AFM spin-orientation for magnetic ground state, producing almost zero magnetic moments (Fig. 2). Proximity with metals at the interfaces prompts spin-polarization in the system due to the transfer of carriers from the delocalized *s-d* hybridized metal layer and the TaAs layer. The ground state magnetic configuration for both the TaAs/Au and TaAs/Ag single interfaces is ferromagnetic (FM). Table 1 lists the magnetic moments, type of doping and ground state magnetic configuration of the interfacial systems.

**Table 1:** Magnetic Moments, nature of doping and ground state configuration of different stack systems.

	System	Magnetic moment (µ_B_)	Nature of doping	Ground state configuration
Single interface	TaAs	0.7520	–	AFM
	Ag–TaAs	2.6760	*n*-type	FM
	Au–TaAs	3.9020	*n*-type	FM
Stacked interface	TaAs	0	–	AFM
	Ag–TaAs	− 0.0013	*p*-type	AFM
	Au–TaAs	− 0.0081	*n*-type	FM

**Figure 4:**
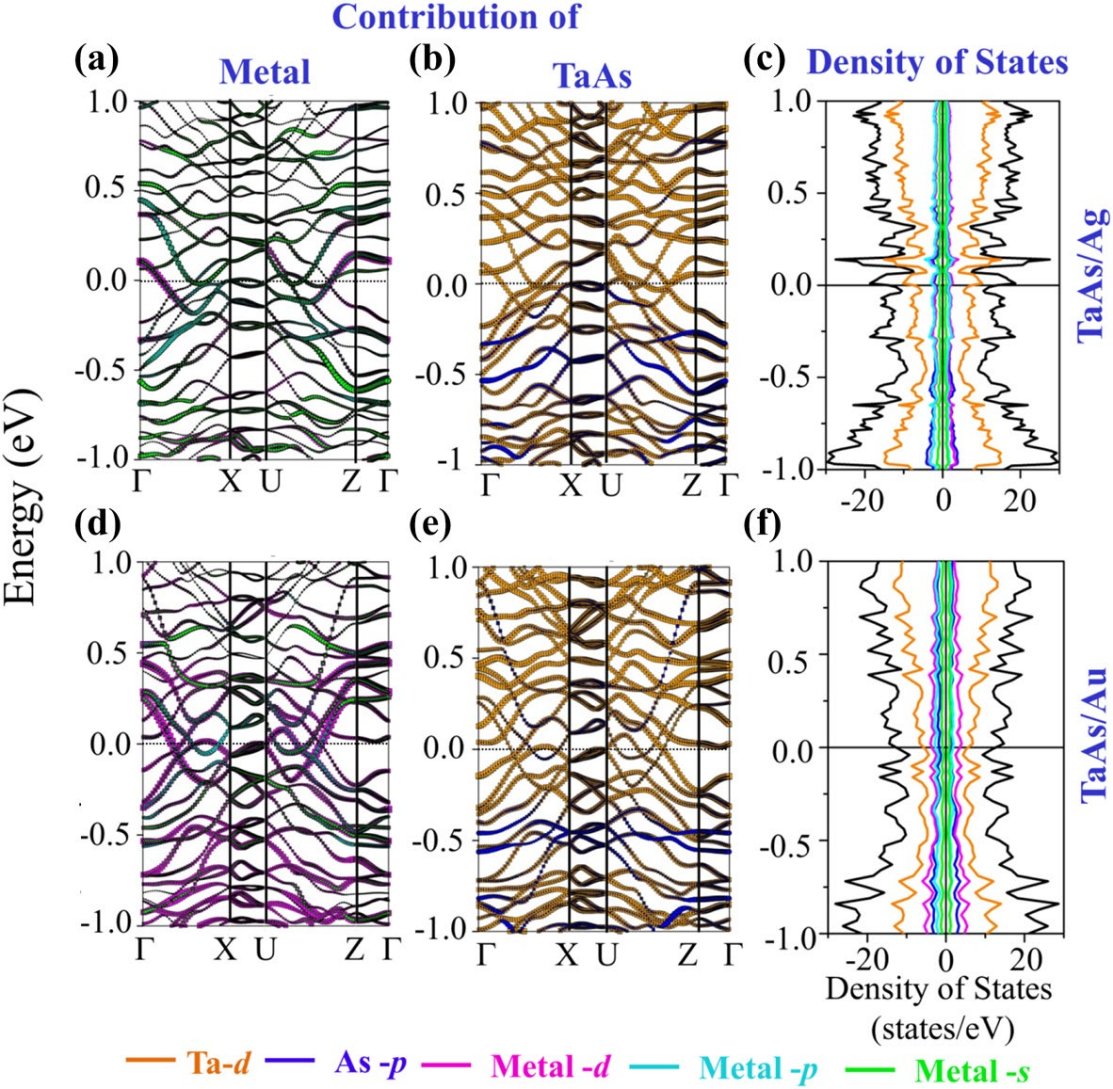
Orbital Projected bands of different systems (**a**) TaAs contribution in TaAs/Ag, (**b**) Ag contribution in TaAs/Ag, and (**c**) the corresponding spin-up and spin-down DOS. Orbital Projected bands of (**d**) TaAs contribution in TaAs/Au, (**e**) Au contribution in TaAs/Au, and (**f**) the corresponding spin-up and spin-down DOS. The color coding for orbital projections in band structure and DOS are: Ta-d—orange, As-p—blue, metal-s—green, metal-p—cyan, metal-d—magenta.

**Figure 5:**
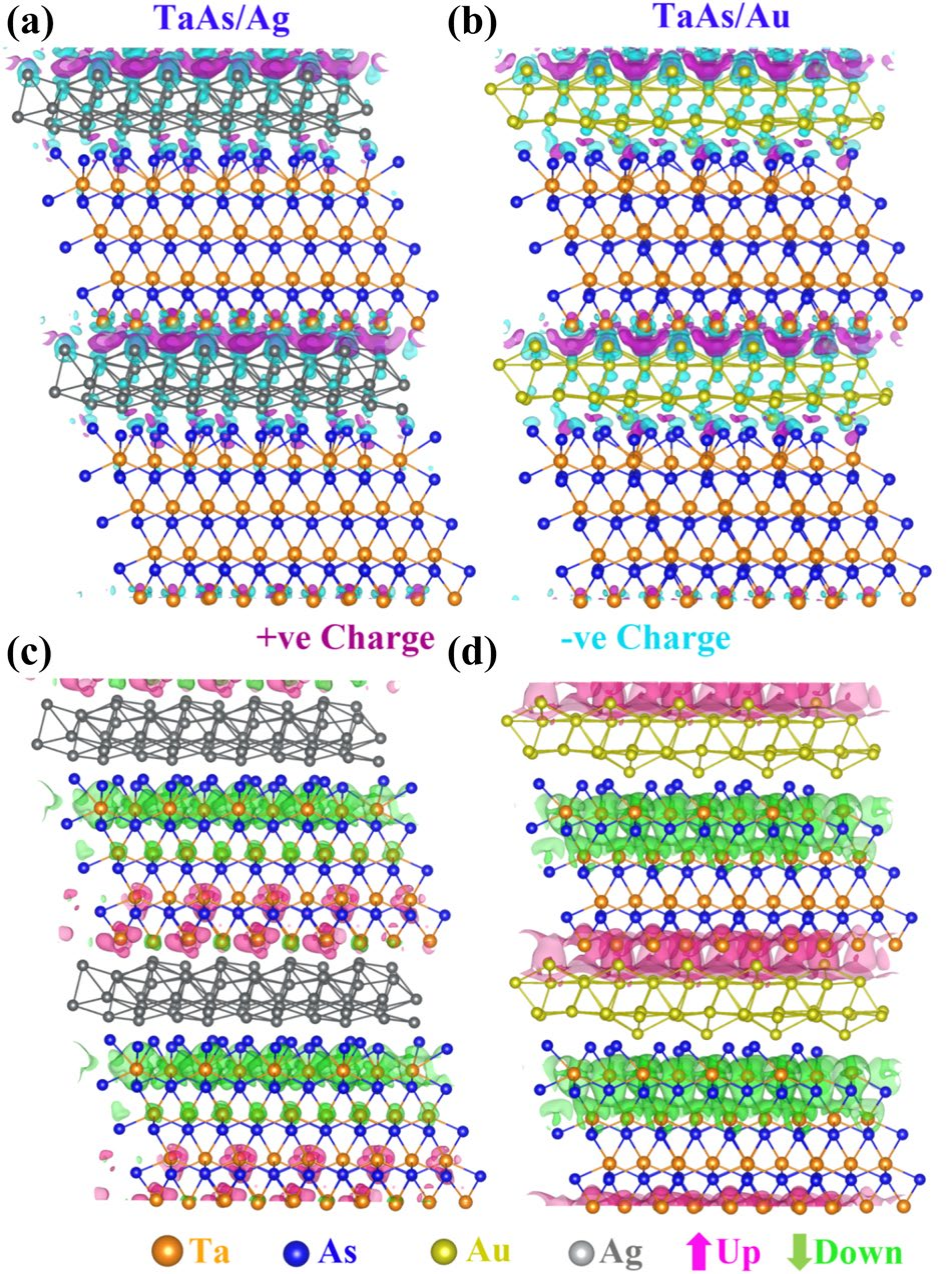
Charge density difference plot of stacked interfaces (**a**) TaAs/Ag and (**b**) TaAs/Au and spin density plot of (**c**) TaAs/Ag and (**d**) TaAs/Au. The color coding for different atoms are: Ta-d—orange, As-p—blue, Ag—grey and Au—yellow. Positive and negative charges are denoted by purple and cyan colour respectively. The spin-up and spin-down spin-densities are designated by magenta and green colours respectively.

Albeit introduction of spin-polarization, the broken inversion symmetry of the interfacial system does not make the presence of the Weyl cones very obvious, due to the presence of huge vacuum slab for the TaAs/Au and TaAs/Ag single interfaces. The confinement effect in the reciprocal space along z-direction leads to the apparent destruction of Weyl feature. The non-degenerate band-crossings and the corresponding nodal features can however be restored upon formation of vertically stacked interfaces like TaAs/Au/TaAs/Au… and TaAs/Ag/TaAs/Ag…. Figure 4 represents the atom-projected band-structures and DOS for the stacked interfaces, the key features of which are listed below:

A. The most fascinating effect of stacked interfaces is the restoration of non-degenerate band-crossings and nodal features along X–U for both the systems, as can be seen from Fig. 4a, b, d and e. The 3D band-crossings are sustained upon application of SOC, as presented in the supplementary figure Fig. S2.
B. The band crossings along X–U are seen to be distributed along the full energy-axis range, as an effect of folding of bands in the BZ of the supercell. With an appropriate doping induced shift of the Fermi-level in the form of applied bias, these systems will be capable of retaining its band-crossing features.
C. The doping nature and the magnetic ground states have undergone a change with respect to the single interfaces, with the TaAs/Ag stacked interface having a *p*-type doping and AFM configuration of the spin-magnetic moments, as can be seen from Table 1. The hole-doped antiferromagnetism in TaAs/Ag system may render it to be a potential correlated system, where holes are doped into the TaAs layer, constituted of less correlated Ta-5*d* levels. This is different from the other hole-doped antiferromagnetic systems like the cuprates and pnictides^24–26,57,58^, where more correlated and localized Cu-3*d* or Fe-3*d* orbitals play a prominent role. For TaAs/Au, the system stabilizes is an *n*-type FM ground state with a small magnetic moment.
D. According to the Kramers theorem, in presence of both TRS and IS, the full band-structure is doubly degenerate at all *k*-points. Thus, by respecting any one of the symmetries and breaking the other, non-degenerate band crossings can be materialized, producing topologically stable *k*-space separated Weyl nodes at the same energy. If both the symmetries are simultaneously broken, the *k*-space separation and thus obtained topological stability of Weyl node remains intact. However, the pairs of Weyl nodes now occur at two different energies, deviating slightly from the nodal semimetal^23^. According to Zyunin et al*.*, application of an external magnetic field along the growth direction of such heterostructure is capable of producing a non-dissipative ground state current^23^. For pristine TaAs, the TRS is respected and broken IS leads to the 3D non-degenerate band crossing under application of SOC. In proximity of Noble metals, the resulting spin-polarization prompts a spontaneous breaking of the time reversal symmetry in this system. Additionally, as a result of broken IS, the band-crossing-induced nodal features along X–U arise. Simultaneous presence of nodal feature and spin-polarization suggests a coexistence of both broken TRS and IS for TaAs/Ag or TaAs/Au stacked interfacial systems^8,23–26^. A closer examination of the nodal points has revealed their occurrence at different energy values. Near *E*_F_, the energy differences between the two nodal points are ~ 0.08 eV and ~ 0.2 eV for GGA and GGA + SOC bands respectively. In addition, the magnetic nature of the system emerges due to the presence of other delocalized bands crossing *E*_F_.
E. The orbital-projected band structures, as presented in Fig. 4a, b, d and e, display a strong mixing of metal *s-d* hybridized bands (magenta and green) with the As-4*p* (blue) levels, due to proximity of the top As-layer with metal layer with a significant DOS at the *E*_F_. The DOS for TaAs/Ag (Fig. 4c) shows sharp peaks near *E*_F_, having contribution from Ta-5*d* (orange) and As-4*p* (blue), whereas Fig. 4f (TaAs/Au) depicts an itinerant electronic nature. The charge density difference (Fig. 5a and (b)) and spin-density plots (Fig. 5c, d) also support the fact of increase of spin-polarization for stacked interfaces with a significantly visible charge-difference density at the metal-semimetal interface and a prominent spin-density in place of the otherwise invisible spin-densities for pristine system.

### Quantum transport for TaAs devices with Au/Ag contacts

**Figure 6:**
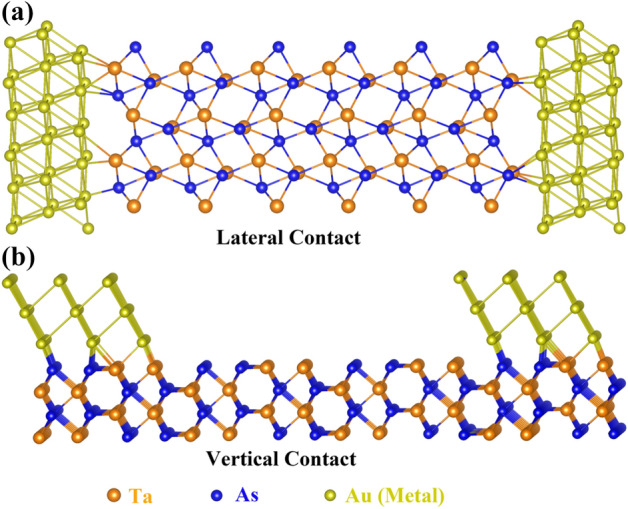
(**a**) Structural representation of (**a**) TaAs with lateral metal contact, and (**b**) TaAs with vertical metal contact.

**Figure 7:**
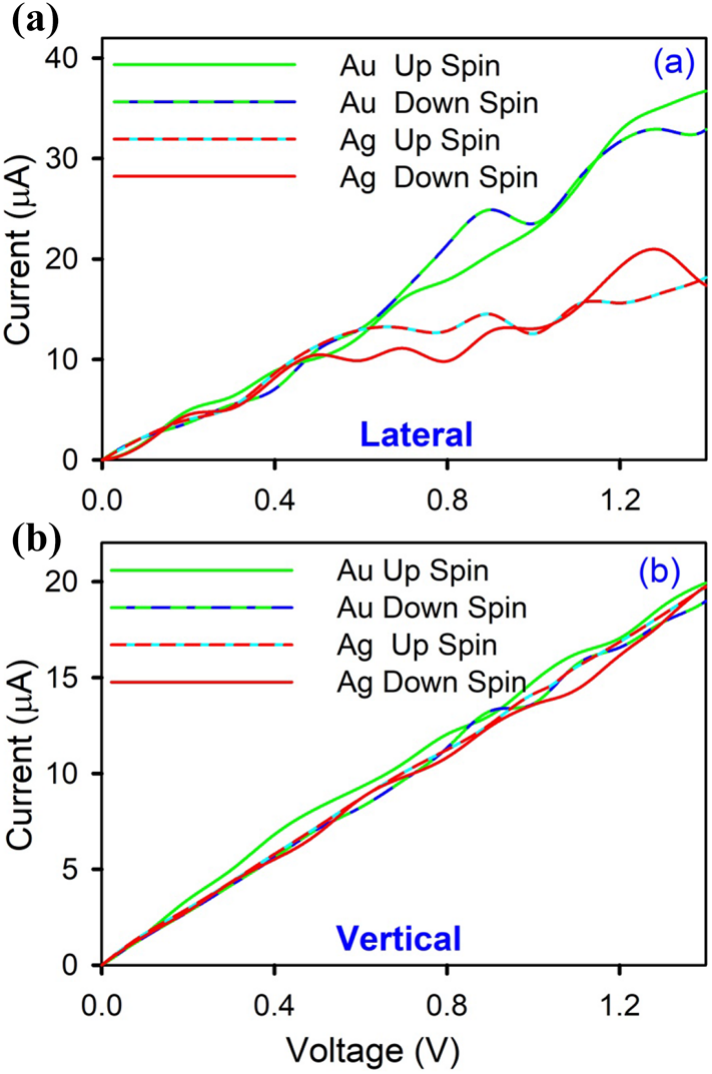
Transport characteristics for (**a**) lateral contacts and (**b**) vertical contact geometries for both TaAs/Ag and TaAs/Au.

**Figure 8:**
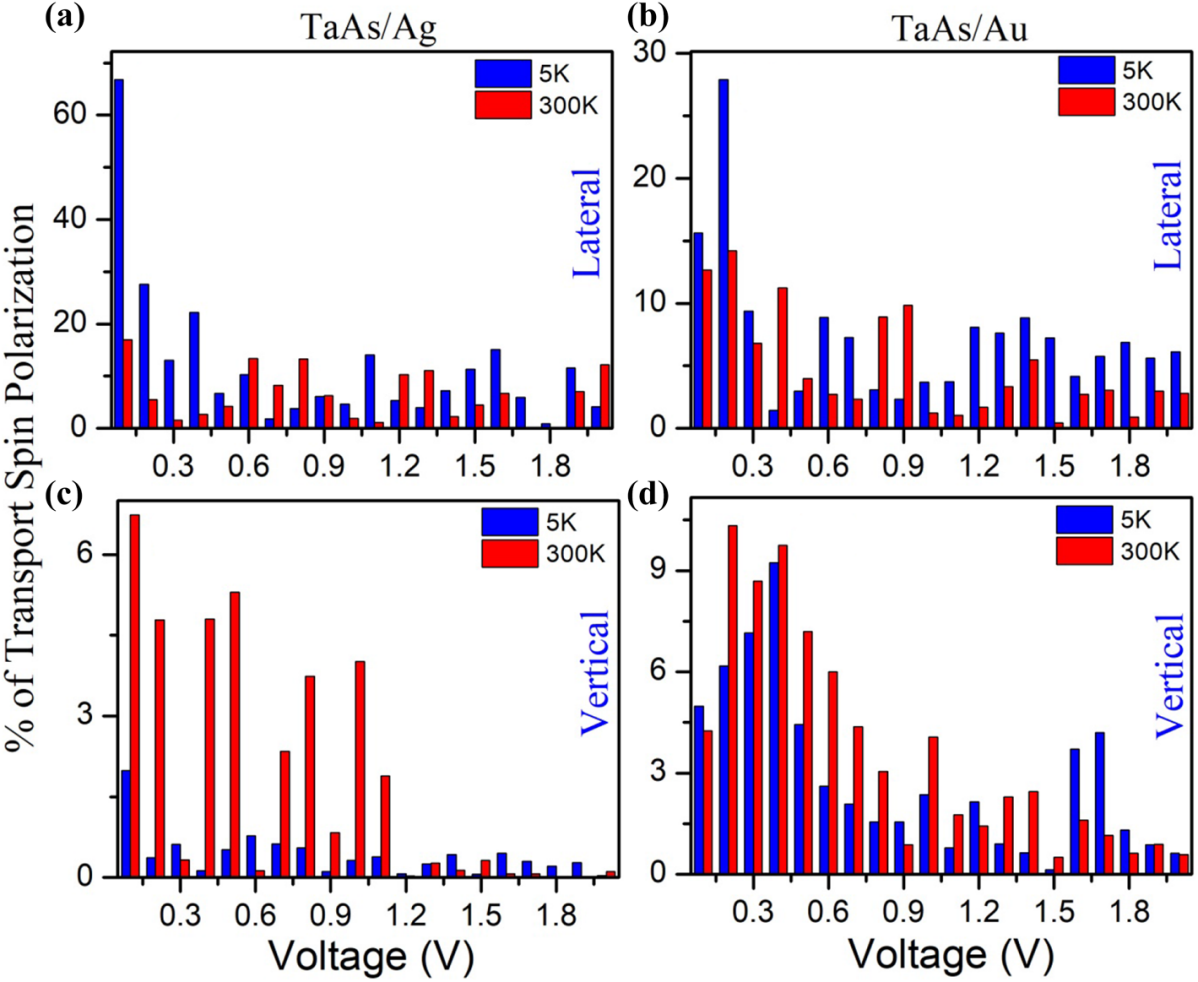
Percentage of transport spin polarization of (**a**) TaAs/Ag lateral, (**b**) TaAs/Au lateral, (**c**) TaAs/Ag vertical and (**d**) TaAs/Au vertical contact geometries.
C. Figure 9 presents the total (up-spin + down-spin) transmission spectra and the corresponding interpolated color-maps at 1 V applied bias on the Γ- centred *K*_A_–*K*_B_ plane *perpendicular to the transport axis*. The lateral contacts have shown a uniform transmission across the transport axis, whereas the vertical ones are having various transmission zones. The average transmission is higher in Ag-contacts for lateral devices, whereas it is almost similar for the vertical contacts for both Ag and Au.

As seen by the point-contact spectroscopic measurements, the transport spin-polarization of TaAs undergoes an increase in presence of Au or Ag tip contacts^38,39^. In this section, we have investigated the transport properties of two probe devices made out of TaAs as channel and Au/Ag as lateral or vertical contacts. The schematic constructions of lateral and vertical contact devices are shown in Fig. 6a and b. The consolidated results of quantum transport calculations and their respective analysis are presented as below:

A. Figure 7 represents the current (*I*) versus voltage (*V*) characteristics for the lateral and vertical contact systems with Au and Ag contacts at room temperatures. In room temperature transport characteristics, transport spin-polarization is more prominent for lateral contacts.
B. In Fig. 8, we have plotted a comparison of the percentage of transport spin polarization for different bias voltages, calculated from its absolute value *P*_*t*_ = (*I*_↑_- *I*_↓_)/( *I*_↑_ + *I*_↓_) for the electron temperatures 5 K and 300 K for both lateral and vertical contact geometry, where *I*_↑_ and *I*_↓_ are the spin-up and spin-down currents respectively. On an average, for both systems, the value of *P*_*t*_ is an order of magnitude more for lateral contacts with a trend of increase in polarization after decreasing temperature. For vertical contacts, on the other hand, *P*_*t*_ at room temperature is more.

**Figure 9:**
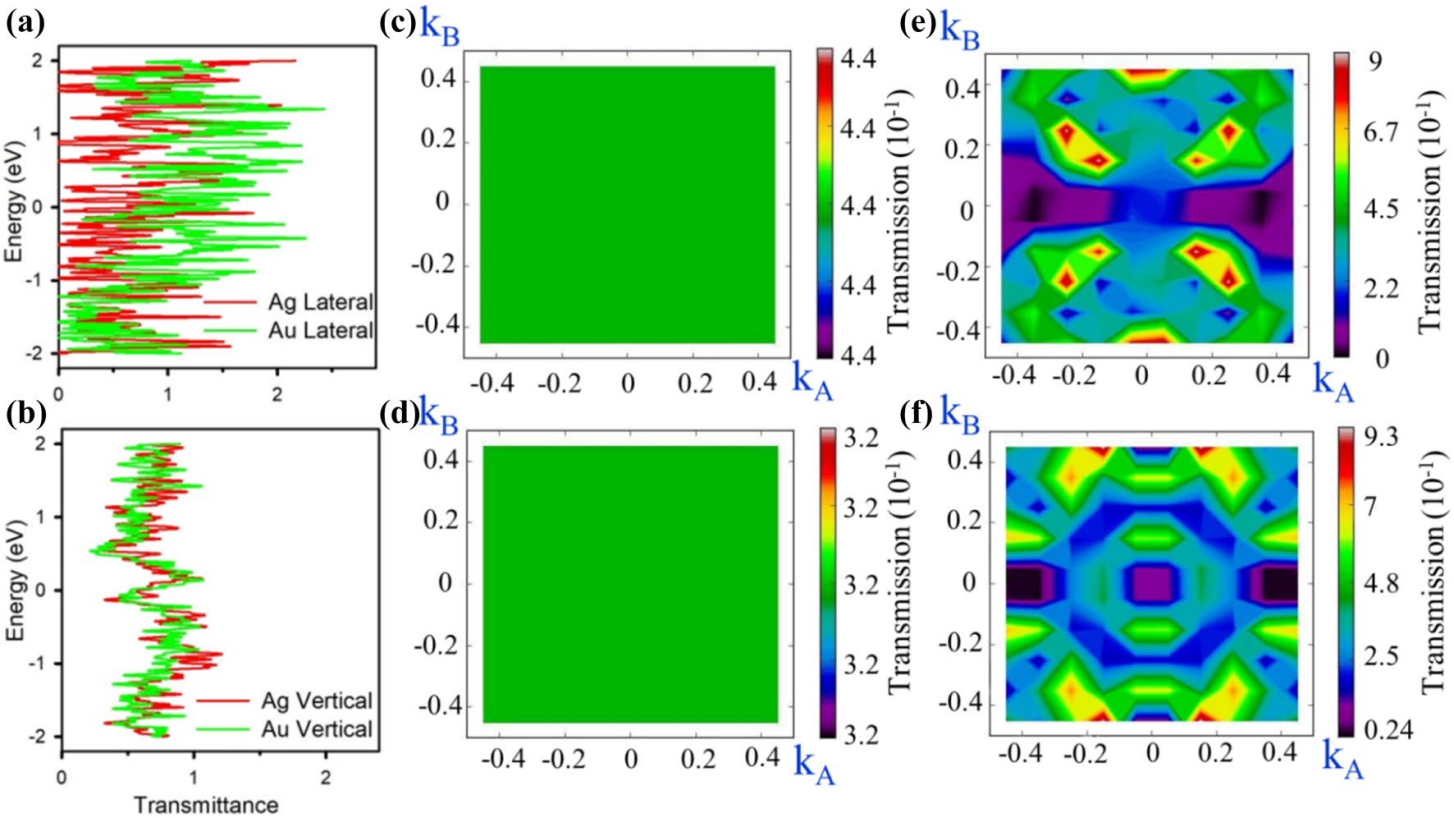
Transmittance of (**a**) Lateral TaAs/Ag and TaAs/Au, (**b**) vertical TaAs/Ag and TaAs/Au, Transmission colour map of (**c**) Ag-Lateral, (**d**) Au-Lateral, (**e**) Ag-vertical and (**f**) Au-vertical.
D. The local density of states (LDOS), presented in Fig. 10, however, shows the difference of transport between the two types of devices *along the device transport axis*. Whereas, the lateral contacts show less interfacial scattering at contacts, implying smoother transmission, the vertical contacts have more scattering near contact boundary. Overall, lateral contacts are more effective in spin-transport in terms of its capability to retain the injected spin-polarization.

**Figure 10:**
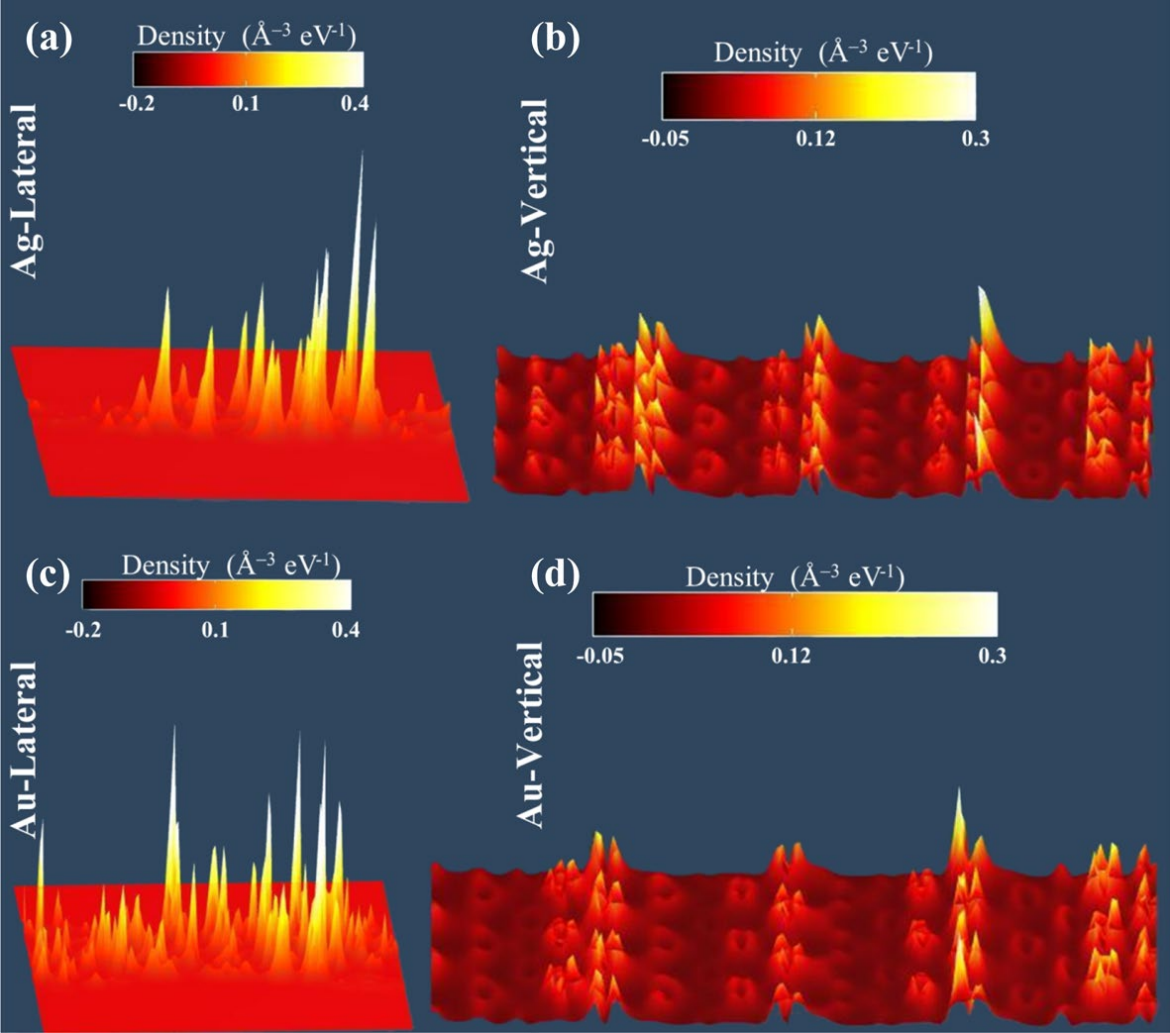
Local density of states (LDOS) for (**a**) Ag-lateral, (**b**) Ag- vertical, (**c**) Au-lateral and (**d**) Au-vertical contact devices.

Thus, while measuring device transport, the types of contact metals and the geometry of contacts have tremendous impact on the spin-polarized device transport.

### Phonon dispersion for stacked interfaces

**Figure 11:**
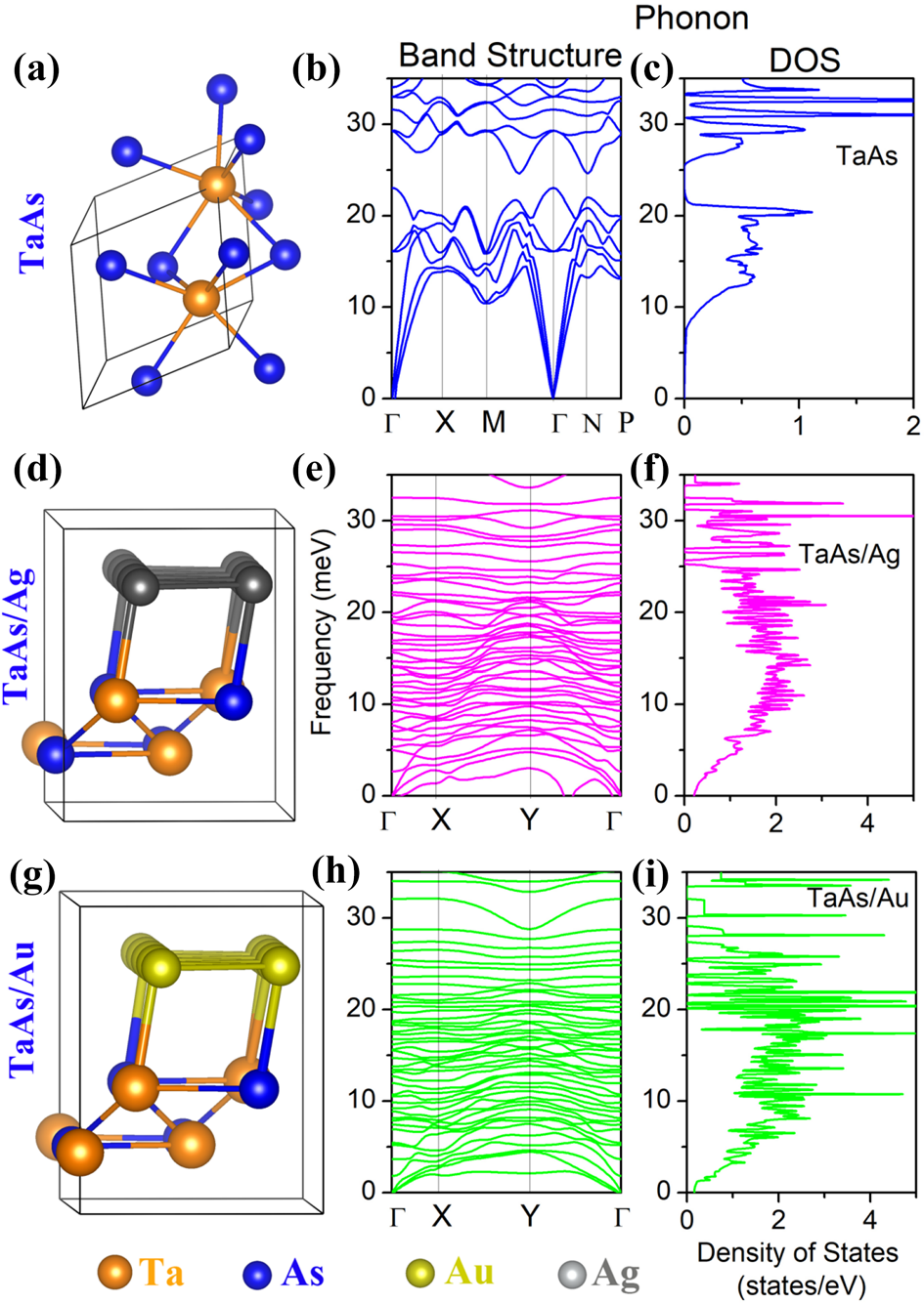
(**a**) Structural representation of TaAs (**b**) corresponding phonon band structure (blue) and (**c**) DOS of TaAs (blue); (**d**) Structural representation of TaAs/Ag, (**e**) the corresponding phonon band structure (magenta) and (**f**) DOS of TaAs/Ag (magenta); (**g**) Structural representation of TaAs/Au (**h**) corresponding phonon band structure (green) and (**i**) DOS of TaAs/Au (green). The color coding for different atoms are: Ta-d—orange, As-p—blue, Ag—grey and Au—yellow.

Since, the lateral contacts with the metal layer bonded with both Ta and As are more promising in producing a better transport spin polarizations, we intend to obtain the nature of phonon dispersion of TaAs in presence of lateral Au or Ag metal layer, where we have created a smaller system resembling the lateral interface as shown in first column of Fig. 11. Corresponding to each structure, the phonon band dispersion and the DOS are plotted in the same row, the essential features of which can be enlisted as:

A. The band dispersion for bulk TaAs (Fig. 11b, blue) resembles with the literature^59,60^, where the well-dispersed acoustic modes, having the most contribution from the vibration of the heavier Ta-ions are separated from the more localized optical counterpart due to vibrations of the lighter As-ions by a band gap of ~ 2.4 meV. Occurrence of phonon band-gaps due to the mass-discrepancy of the contributing ions is well-known^61^.
B. In presence of lateral interfaces with Ag (Fig. 11e, magenta) and Au (Fig. 11h, green), the band-gap disappears with a significant increase of the density of localized phonon bands in the acoustic frequency range, as can be seen from the second column of Fig. 11. In addition, for TaAs/Ag system, there is presence of soft phonons with imaginary frequencies, which may be a signature of presence of instabilities in the respective system.
C. The enhanced density of acoustic phonon bands also induces a corresponding increase of phonon DOS for both of the metal-stackings, viz. Ag (Fig. 11f, magenta) and Au (Fig. 11i, green). The phonon DOS for both of these cases almost doubles in the acoustic range in association with the localized peaks. However, any phonon-mediated correlated phenomena will also depend on the electronic deformation potential at those energy ranges.

Thus, in presence of metals, there is an eloquent impact on the phonon dispersion and densities of TaAs, suggesting emergence of phonon-induced correlated behaviour within the system.

## Conclusion

In summary, we have investigated the macroscopic single and stacked interfaces of the Weyl semimetal system TaAs with two noble metals Au and Ag to study the proximity effects at the noble metal-semimetal interfaces. (1) The band-structures for stacked interfaces have manifested a simultaneous breaking of both inversion symmetry and time-reversal symmetry for TaAs/Ag and TaAs/Au systems. (2) Whereas, pristine TaAs is non-magnetic, TaAs/Au and TaAs/Ag show electron-doped FM and hole doped AFM ground-states. (3) Transport properties of lateral and vertical contact geometries have significant impact of transport spin-polarization both at room temperature and at low temperatures. Lateral contacts, on an average, have more uniform transport and more transport spin-polarization with lowering of temperature and thus are more effective in spin-transport. (4) The phonon-dispersion for the lateral-contact systems indicates a disappearing phonon band gap and an increase of acoustic phonon density of states.

## Supplementary information

Supplementary Information.

## References

[1] Lv B (2015). Experimental discovery of Weyl semimetal TaAs. Phys. Rev. X.

[2] Xu S-Y (2015). Experimental discovery of a topological Weyl semimetal state in TaP. Sci. Adv..

[3] Weyl H (1929). Gravitation and the electron. Proc. Natl. Acad. Sci. USA.

[4] Wang Z (2012). Dirac semimetal and topological phase transitions in A 3 Bi (A= Na, K, Rb). Phys. Rev. B.

[5] Wang Z, Weng H, Wu Q, Dai X, Fang Z (2013). Three-dimensional Dirac semimetal and quantum transport in Cd 3 As 2. Phys. Rev. B.

[6] Hasan MZ, Kane CL (2010). Colloquium: topological insulators. Rev. Mod. Phys..

[7] Qi X-L, Zhang S-C (2011). Topological insulators and superconductors. Rev. Mod. Phys..

[8] Yan B, Felser C (2017). Topological materials: Weyl semimetals. Annu. Rev. Condens. Matter Phys..

[9] Rao S (2016). Weyl Semi-Metals: A Short Review. J. Indian Inst. Sci..

[10] Weyl H (1929). Elektron und gravitation. I.. Z. Phys. Had. Nucl..

[11] Wan X, Turner AM, Vishwanath A, Savrasov SY (2011). Topological semimetal and Fermi-arc surface states in the electronic structure of pyrochlore iridates. Phys. Rev. B.

[12] Balents L (2011). Weyl electrons kiss. Physics.

[13] Turner AM, Vishwanath A, Head CO (2013). Beyond band insulators: topology of semimetals and interacting phases. Topol. Insul..

[14] Vafek O, Vishwanath A (2014). Dirac fermions in solids: from high-T c cuprates and graphene to topological insulators and Weyl semimetals. Annu. Rev. Condens. Matter Phys..

[15] Young SM (2012). Dirac semimetal in three dimensions. Phys. Rev. Lett..

[16] Yang B-J, Nagaosa N (2014). Classification of stable three-dimensional Dirac semimetals with nontrivial topology. Nat. Commun..

[17] Huang S-M (2015). Theoretical discovery/prediction: Weyl semimetal states in the TaAs material (TaAs, NbAs, NbP, TaP) class. Nat. Commun..

[18] Chang G (2015). Quasi-particle interferences of the Weyl semimetals TaAs and NbP. Phys. Rev. Lett..

[19] Huang X (2015). Observation of the chiral-anomaly-induced negative magnetoresistance in 3D Weyl semimetal TaAs. Phys. Rev. X.

[20] Weng H, Fang C, Fang Z, Bernevig BA, Dai X (2015). Weyl semimetal phase in noncentrosymmetric transition-metal monophosphides. Phys. Rev. X.

[21] Liu Z (2014). Discovery of a three-dimensional topological Dirac semimetal, Na3Bi. Science.

[22] Burkov AA, Balents L (2011). Weyl semimetal in a topological insulator multilayer. Phys. Rev. Lett..

[23] Zyuzin AA, Wu S, Burkov AA (2012). Weyl semimetal with broken time reversal and inversion symmetries. Phys. Rev. B.

[24] Muechler L (2020). Emerging chiral edge states from the confinement of a magnetic Weyl semimetal in Co3Sn2S2. Phys. Rev. B.

[25] Xu Q (2018). Topological surface Fermi arcs in the magnetic Weyl semimetal Co3Sn2S2. Phys. Rev. B.

[26] Liu E (2018). Giant anomalous Hall effect in a ferromagnetic kagome-lattice semimetal. Nat. Phys..

[27] Xu G, Weng H, Wang Z, Dai X, Fang Z (2011). Chern semimetal and the quantized anomalous hall effect in HgCr2Se4. Phys. Rev. Lett..

[28] Dirac PAM (1931). Quantised singularities in the electromagnetic field. Proc. R. Soc. Lond..

[29] Hooft TG (1974). Magnetic monopoles in unified theories. Nucl. Phys. B.

[30] Polyakov AM (1996). 30 Years of the Landau Institute—Selected Papers.

[31] Fang Z (2003). The anomalous hall effect and magnetic monopoles in momentum space. Science.

[32] Parameswaran S, Grover T, Abanin D, Pesin D, Vishwanath A (2014). Probing the chiral anomaly with nonlocal transport in three-dimensional topological semimetals. Phys. Rev. X.

[33] Nielsen HB, Ninomiya M (1983). The Adler–Bell–Jackiw anomaly and Weyl fermions in a crystal. Phys. Rev. B.

[34] Adler SL (1969). Axial-vector vertex in spinor electrodynamics. Phys. Rev..

[35] Bell JS, Jackiw R (1969). A PCAC puzzle: π 0→ γγ in the σ-model. Nuovo Cimento A.

[36] Stone M, Gaitan F (1987). Topological charge and chiral anomalies in fermi superfluids. Ann. Phys..

[37] Volovik G (1987). Peculiarities in the dynamics of superfluid 3He-A: analog of chiral anomaly and of zero-charge. Sov. Phys. JETP Lett..

[38] Aggarwal L (2016). Unconventional superconductivity at mesoscopic point contacts on the 3D Dirac semimetal Cd3As2. Nat. Commun..

[39] Aggarwal L (2017). Mesoscopic superconductivity and high spin polarization coexisting at metallic point contacts on Weyl semimetal TaAs. Nat. Commun..

[40] Wang H (2016). Tip induced unconventional superconductivity on Weyl semimetal TaAs. Sci. Bull..

[41] Wang, H. *et al.* Reply to Comment on “Tip induced unconventional superconductivity on Weyl semimetal TaAs”. arXiv preprint arXiv:1607.02886 (2016).

[42] Gayen, S., Aggarwal, L. & Sheet, G. Comment on “Tip induced unconventional superconductivity on Weyl semimetal TaAs”. arXiv preprint arXiv:1607.01405 (2016).

[43] Wang P (2017). A theory of nonequilibrium steady states in quantum chaotic systems. J Stat. Mech. Theory Exp..

[44] Somvanshi D (2017). Nature of carrier injection in metal/2D-semiconductor interface and its implications for the limits of contact resistance. Phys. Rev. B.

[45] Popov I, Mantega M, Narayan A, Sanvito S (2014). Proximity-induced topological state in graphene. Phys. Rev. B.

[46] Wu G (2013). Tuning the vertical location of helical surface states in topological insulator heterostructures via dual-proximity effects. Sci. Rep..

[47] Kresse G, Furthmüller J (1996). Efficient iterative schemes for ab initio total-energy calculations using a plane-wave basis set. Phys. Rev. B.

[48] Kresse G, Joubert D (1999). From ultrasoft pseudopotentials to the projector augmented-wave method. Phys. Rev. B.

[49] Ernzerhof M, Scuseria GE (1999). Assessment of the Perdew–Burke–Ernzerhof exchange-correlation functional. J Chem. Phys..

[50] Grimme S, Antony J, Ehrlich S, Krieg H (2010). A consistent and accurate ab initio parametrization of density functional dispersion correction (DFT-D) for the 94 elements H-Pu. J. Chem. Phys..

[51] Monkhorst HJ, Pack JD (1976). Special points for Brillouin-zone integrations. Phys. Rev. B.

[52] Shewchuk JR (1994). An Introduction to the Conjugate Gradient Method Without the Agonizing Pain.

[53] Smidstrup S (2019). QuantumATK: an integrated platform of electronic and atomic-scale modelling tools. J. Phys. Condens. Matter.

[54] Maji TK (2019). Intricate modulation of interlayer coupling at the graphene oxide/MoS e 2 interface: application in time-dependent optics and device transport. Phys. Rev. B.

[55] Lee C-C (2015). Fermi surface interconnectivity and topology in Weyl fermion semimetals TaAs, TaP, NbAs, and NbP. Phys. Rev. B.

[56] Huang S-M (2015). A Weyl Fermion semimetal with surface Fermi arcs in the transition metal monopnictide TaAs class. Nat. Commun..

[57] Wang M (2013). Doping dependence of spin excitations and its correlations with high-temperature superconductivity in iron pnictides. Nat. Commun..

[58] da Silva Neto EH (2014). Ubiquitous interplay between charge ordering and high-temperature superconductivity in cuprates. Science.

[59] Buckeridge J, Jevdokimovs D, Catlow C, Sokol A (2016). Bulk electronic, elastic, structural, and dielectric properties of the Weyl semimetal TaAs. Phys. Rev. B.

[60] Ouyang T, Xiao H, Tang C, Hu M, Zhong J (2016). Anisotropic thermal transport in Weyl semimetal TaAs: a first principles calculation. Phys. Chem. Chem. Phys..

[61] Kim H (2015). First-principles calculations of the lattice instability and the symmetry-lowering modulation of PtSi. J Kor. Phys. Soc..

