# Supplementary material for: Broken symmetries and the related interface-induced effects at Weyl-system TaAs in proximity of noble metals
Source: Sci Rep. 2020 Sep 2;10:14438. doi: 10.1038/s41598-020-71494-w (PMC7468271; doi:10.1038/s41598-020-71494-w)
Supplement: Supplementary file 1 — Supplementary Information. [file 41598_2020_71494_MOESM1_ESM.docx]

*Supplementary Article for*

**Broken Symmetries and the Related Interface-induced Effects at Weyl-system TaAs in Proximity of Noble Metals**

Tuhin Kumar Maji^1^, Kumar Vaibhav^2^, Samir Kumar Pal^1^ and Debjani Karmakar^3,^*

*^1^Department of Chemical Biological and Macromolecular Sciences, S.N. Bose National Centre for Basics Sciences, Salt Lake, Sector 3, Kolkata 700106, India*

*^2^Computer Division, Bhabha Atomic Research Centre, Trombay, Mumbai 400085, India*

*^3^Technical Physics Division, Bhabha Atomic Research Centre, Trombay, Mumbai 400085, India*

*Corresponding Author: Debjani Karmakar: [debjan@barc.gov.in](mailto:debjan@barc.gov.in)


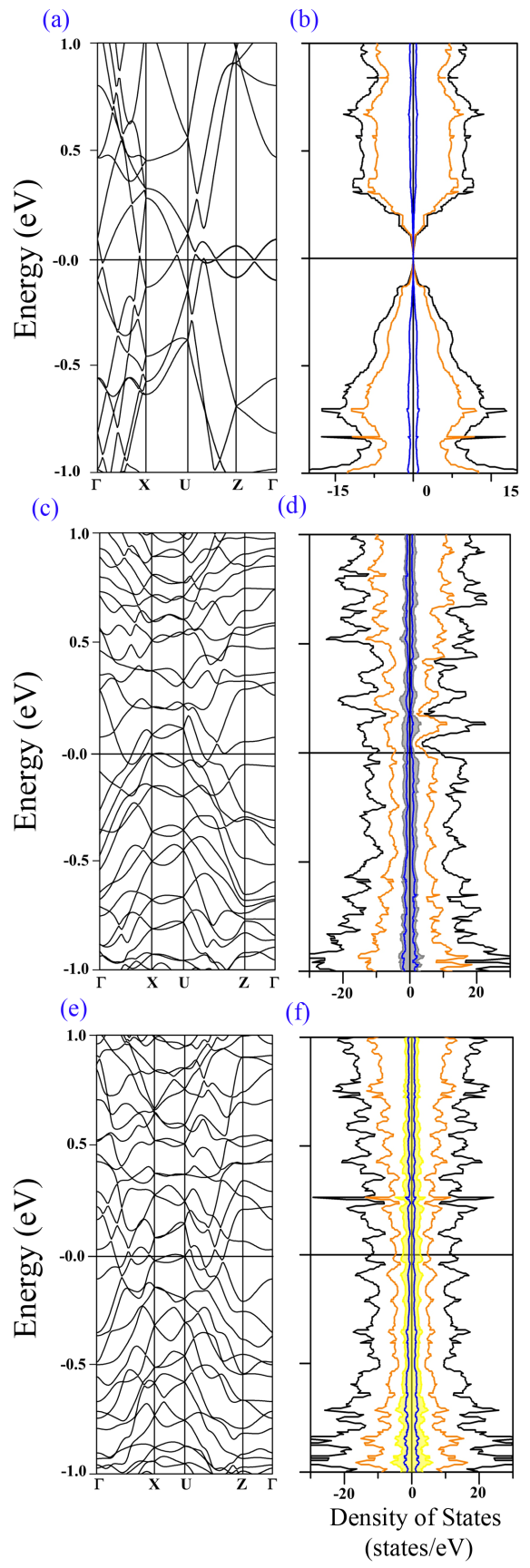


**Figure S1:** GGA+U (a) band structure and (b) orbital projected DOS of pristine TaAs, GGA+U c) band structure and d) orbital projected DOS of TaAs/Ag stacked interface and GGA+U e) band structure and f) orbital projected DOS of TaAs/Au stacked interface. The Ta-d and As-p orbital characters are denoted by orange and blue colours respectively.


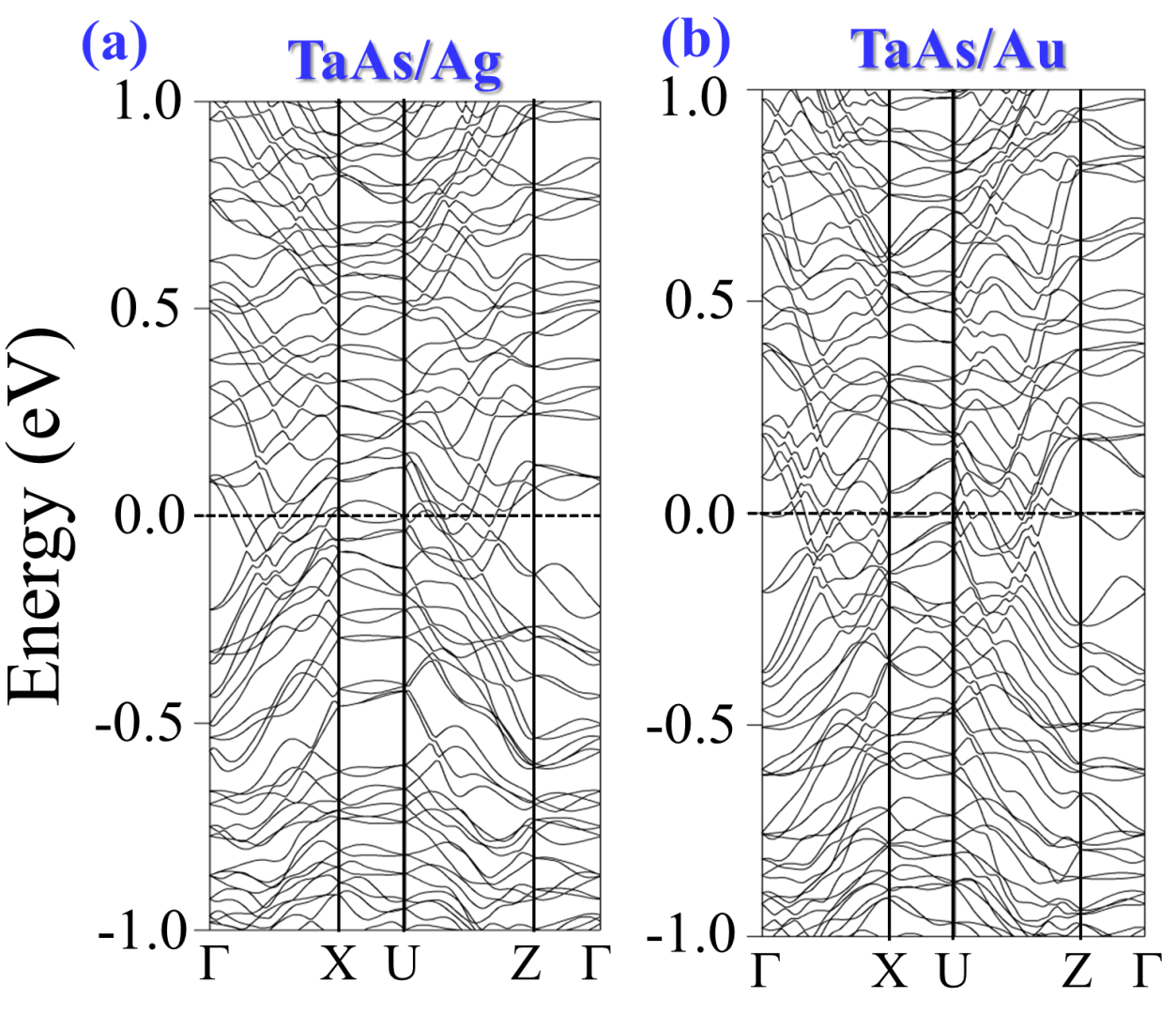


**Figure S2:** GGA + SOC bands for a) TaAs/Ag and b) TaAs/Au stacked interfaces.
